# Reliability of Rehabilitative Musculoskeletal Sonography for Measuring the Visible Cross-Sectional Area of Suboccipital Muscles

**DOI:** 10.7759/cureus.68772

**Published:** 2024-09-06

**Authors:** Pezhman Masoudi, Noureddin Karimi, Iraj Abdollahi, Saeideh Moravej, Arash Tahamtan

**Affiliations:** 1 Physical Therapy, University of Social Welfare and Rehabilitation Sciences, Tehran, IRN; 2 Medicine, Islamic Azad University of Medical Sciences, Tehran, IRN

**Keywords:** obliquus capitis inferior, rectus capitis posterior major, rectus capitis posterior minor, suboccipital muscle sonography, upper cervical muscles

## Abstract

Aims

The primary aim of this study is to assess the reliability of rehabilitative sonography as a non-invasive imaging technique for measuring the visible cross-sectional area (CSA) of the deep suboccipital muscles.

Objectives

Objectives involve determining both the intra-rater and inter-rater reliability of sonographic measurements to ensure consistent and reproducible results across different sessions and examiners. The ultimate goal is to validate rehabilitative sonography as a reliable tool for clinical and research applications in the assessment and management of musculoskeletal (MSK) conditions involving the deep suboccipital muscles.

Design and setting

Seventeen participants, including nine women and eight men without neck or vertebral pain, were evaluated using MSK sonography. The visible CSA of suboccipital muscles was assessed using real-time B-mode sonography in a sitting position with a linear probe (5 cm, 7.5 MHz) aligned perpendicularly to the muscle fibers of the rectus capitis posterior minor (RCPM) in one sonogram and the obliquus capitis inferior (OCI) and rectus capitis posterior major (RCPMJ) in another. Two experienced examiners performed sonography measurements, and the procedure was repeated one hour and again one day later.

Results

Intra-class correlation coefficient (ICC) values for Examiner 1 were 0.84 and 0.79 for RCPM, 0.84 and 0.71 for RCPMJ, and 0.86 and 0.77 for OCI at the one-hour and one-day repetitions, respectively. ICC values for Examiner 2 were 0.86 and 0.77 for RCPM, 0.77 and 0.86 for RCPMJ, and 0.61 and 0.64 for OCI at the one-hour and one-day repetitions, respectively. Inter-rater ICC values were 0.77 for RCPM, 0.82 for RCPMJ, and 0.68 for OCI.

Standard error of measurement (SEM) values for Examiner 1 were 11.63 and 13.92 mm² for RCPM, 10.30 and 7.91 mm² for RCPMJ, and 22.84 and 34.61 mm² for OCI. SEM values for Examiner 2 were 11.82 and 13.40 mm² for RCPM, 11.91 and 7.04 mm² for RCPMJ, and 39.20 and 37.73 mm² for OCI. SEM values between examiners were 13.74 mm² for RCPM, 10.36 mm² for RCPMJ, and 36.03 mm² for OCI.

Conclusions

These findings suggest that sonography is a reliable method for measuring the CSA of the RCPM, RCPMJ, and OCI muscles, provided that the examiners are well-trained and consistent in their techniques and that the average of three measurements is used.

## Introduction

The rectus capitis posterior minor (RCPM), rectus capitis posterior major (RCPMJ), and obliquus capitis inferior (OCI) are part of the suboccipital muscle group and are located in the upper cervical region, playing roles in head and neck movement. The RCPM muscle extends from the posterior tubercle of the atlas (C1) to the medial part of the inferior nuchal line of the occipital bone. In contrast, the RCPMJ muscle originates from the spinous process of the axis (C2) and attaches to the lateral part of the inferior nuchal line. The OCI muscle runs from the spinous process of the axis (C2) to the transverse process of the atlas (C1) [1,2].

Beyond their roles as prime movers, these muscles are integral to proprioception, particularly in the upper cervical region. Studies have shown that they are connected to saccadic eye movements [3] and are major proprioceptive sources in the neck. Dysfunction in the proprioceptive feedback from these muscles to the brain can lead to an inability to organize the head and neck position relative to each other, potentially resulting in headaches and dizziness [4-6]. The RCPM muscle, in particular, plays a critical role in head movement and stability by attaching to the myodural bridge, a connective tissue structure that links the dura mater of the spinal cord to the muscle fibers. This unique attachment allows the RCPM to influence the tension and movement of the dura mater, potentially affecting cerebrospinal fluid (CSF) flow. Through its connection with the myodural bridge, the RCPM muscle can modulate intracranial pressure and CSF dynamics, contributing to the overall regulation of the central nervous system [7,8].

Given the proximity of the suboccipital muscles to neural structures such as the vagus nerve, spinal accessory nerve, and greater occipital nerve, some studies suggest possible theoretical involvement of these muscles, in conditions like occipital neuralgia, secondary hypertension, and autonomic dysfunctions, which should be confirmed with future studies [9,10].

Musculoskeletal (MSK) sonography is a valuable tool for diagnosing and managing MSK conditions, particularly when performed by experienced practitioners. Since its inception in the mid-20th century, MSK ultrasound has undergone significant technological and clinical advancements, becoming an indispensable diagnostic tool in many areas of medicine. It is particularly valued for its ability to provide real-time, non-invasive imaging of the MSK system. Despite its advantages, such as time and cost efficiency, MSK sonography has some limitations, including a limited field of view, which may be insufficient for assessing large areas or multiple joints in a single scan. Nonetheless, it remains a standard diagnostic tool in many clinical practices, especially for evaluating soft tissue injuries, guiding injections, and assessing joint and muscle pathology [11,12].

Given the importance of the deep suboccipital muscles in the head and neck region, clinicians require accessible, cost-effective quantitative measurements of these muscles to enhance the treatment of related disorders. MSK sonography, as a readily available tool, offers a potential solution. Therefore, this study aims to evaluate the reliability of rehabilitative sonography in measuring the visible cross-sectional area (CSA) of the deep suboccipital muscles as a quantitative tool for muscle evaluation.

## Materials and methods

Study design and participants

Seventeen pain-free, healthy participants, including nine women and eight men aged between 22 and 65, were evaluated. The inclusion criteria for the study were: no significant or chronic pain in the cervical, thoracic, and lumbar regions in the past two years, and the participants' agreement to participate in the study. The exclusion criteria included any bothersome, lasting pain in the vertebrae over the past two years; any surgical procedure on the vertebral column; any history of fractures in nearby areas such as the thorax, scapula, humerus, and clavicle, which could potentially change the myofascial structure and physiology by inducing fascial distortions [13,14]; vertebral column deviations in the frontal and sagittal planes, such as scoliosis, forward head posture, and kyphosis, which may alter the myofascial structure and physiology [15]; systemic or neurogenic diseases that may directly influence the muscular structures; and any eye problems that could alter the activation of the suboccipital muscles, as suggested by some studies [16,17]. The maximal force a muscle can produce is an important indicator of its activity and performance. The CSA of a muscle, which quantifies echogenicity and size, can significantly predict its functional capacity [18,19]. In this study, we employed musculoskeletal ultrasound (MSK-US) imaging as a diagnostic tool to assess the visible CSA of three suboccipital muscles in the upper cervical region: RCPM, RCPMJ, and OCI. The focus was to evaluate the sonographic reliability of these measurements. The reliability of these sonographic measurements was determined using the intra-class correlation coefficient (ICC), the standard error of measurement (SEM), and minimal detectable changes (MDC). These metrics provided insights into the consistency and precision of the ultrasound measurements across different examiners and testing sessions. Informed consent was obtained from all participants, who were informed of their right to withdraw from the study at any stage. The study was approved by the relevant Ethics Committee, with approval code IR.USWR.REC.1401.237.

MSK rehabilitation sonography

**Figure 1:**
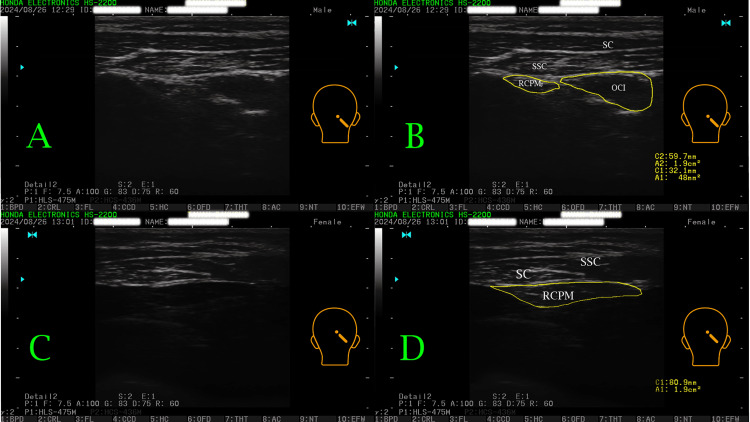
Sonogram of RCPM, RCPMJ, and OCI (A) OCI and RCPMJ sonograms without area measurements. (B) OCI and RCPMJ sonograms with yellow borders outlining the area measurements, including measurement values. (C) RCPM sonogram without area measurement. (D) RCPM sonogram with a yellow border outlining the area measurement, including measurement values. RCPM: rectus capitis posterior minor; RCPMJ: rectus capitis posterior major; OCI: obliquus capitis inferior

Sonography was performed using the HONDA HS-2200 rehabilitative sonography device (Honda Electronics Co., Ltd., Japan) in real-time B-mode recording with 7.5 MHz and a 5 cm linear probe. Participants were seated in a neutral position, with head and cervical alignment traced using the GPS 400 (Global Postural System, Chinesport, Italy). A lined paper was placed on the wall in front of them. Participants found their neutral head position, aligned it with a line on the wall, and maintained this position during the procedure. The ultrasound probe was aligned with the axis of the OCI muscle, from the spinous process of C2 to the transverse process of C1, and rotated about 70 degrees perpendicular to the muscle fibers. The best border-lined sonogram for both OCI and RCPMJ muscles was achieved. In another attempt, the spinous process of C2 was identified by manual palpation. The ultrasound probe was then moved laterally until the lamina of C2 was visualized. From this position, the probe was moved upward to locate the lamina of C1, and then slightly medially to identify the posterior arch of C1. The probe was smoothly adjusted to achieve the best border-lined sonogram of the RCPM. Participants were asked to move their eyes laterally to better identify muscle borders. After this, using the device measurement tools for area/circumference, the visible CSA was measured (Figure 1). The procedure was repeated one hour and one day later for 17 participants to assess the ICC and SEM [10,20].

Examiners

Two qualified and certified examiners conducted the sonography measurements. Participants were randomly assigned to two groups of 10 participants and one residual group of seven. Names were recorded on paper and distributed to each examiner. Each examiner assessed their assigned group with intervals of one hour and one day between measurements. After the initial measurements, participants were exchanged between the examiners for further evaluation, including those in the residual group. All measurements were conducted under similar conditions to ensure consistency between examiners.

Examiner training

Examiner 1 had four years of experience in MSK sonography, while Examiner 2 had two years of experience. Both examiners had been involved in various MSK sonography research projects over the past two years. Their evaluations were supervised and reviewed by a medical sonography specialist to ensure accuracy.

## Results

In this study, two examiners conducted three sonography measurements: an initial measurement, followed by additional measurements at one hour and one day later for RCPM, RCPMJ, and OCI muscles. Seventeen participants were included, with a mean age of 42.47 and a mean BMI of 25.24. The Shapiro-Wilk test for normality confirmed a normal distribution for the contextual variables (see Table 1). The ICC, SEM, and MDC were calculated using Statistical Package for the Social Sciences (IBM SPSS Statistics for Windows, IBM Corp., Version 25.0, Armonk, NY) with a two-way mixed-effects model focused on consistency, to evaluate whether the raters’ scores for the same group of subjects are correlated in an additive manner [21]. SEM provides an estimate of the amount of error associated with an individual's score on a test or measurement tool. It reflects the precision of measurement by indicating how repeated measures of a person on the same instrument tend to be distributed around the "true" score. SEM is crucial for understanding the reliability of measurement instruments, as it helps gauge how much-observed scores fluctuate due to measurement errors. MDC, also known as the smallest real difference, is a statistically derived value that indicates the smallest amount of change in a measurement that exceeds the measurement error's threshold of variability. It represents the smallest change that cannot be attributed to variations in measurement methods or natural fluctuations in performance, ensuring that observed changes are due to actual improvements or deteriorations in the parameter being measured. Table 2 represents the repeated measurements for three suboccipital muscles, conducted by each examiner at three different time points.

**Table 1:** Demographic details of the participants * Frequency (percentage), ** χ²

Metrics		Mean ± SD	Median	Range	Statistics (Normality Test)	Sig.
Age		42.47 ± 14.07	40.0	23.0-65.0	0.908	0.094
BMI		25.24 ± 3.49	24.9	22.5-36.3	0.952	0.487
Sex	Female	52.94%*	-	-	1.231**	0.18
	Male	47.06%*	-	-		

**Table 2:** Mean ± SD for initial, one-hour, and one-day repeated muscle measurements by each examiner Each measurement at the initial, one-hour, and one-day time points included three attempts, with the average taken as the final value. RCPM: rectus capitis posterior minor; RCPMJ: rectus capitis posterior major; OCI: obliquus capitis inferior

Examiner 1 Measurement/Muscle	Mean ± SD	Min-Max	Examiner 2 Measurement/Muscle	Mean ± SD	Min-Max
Initial RCPM	140.24 ± 26.48	94-190	Initial RCPM	144.12 ± 23.99	100-180
One-Hour RCPM	132.59 ± 24.88	84-170	One-Hour RCPM	135.00 ± 25.25	95-180
One-Day RCPM	147.06 ± 25.68	100-200	One-Day RCPM	139.41 ± 22.21	100-170
Initial RCPMJ	97.41 ± 26.22	49-150	Initial RCPMJ	81.59 ± 14.26	50-100
One-Hour RCPMJ	93.59 ± 20.71	62-140	One-Hour RCPMJ	80.88 ± 10.74	65-110
One-Day RCPMJ	88.76 ± 18.11	50-120	One-Day RCPMJ	82.29 ± 17.71	41-110
Initial OCI	235.29 ± 60.74	130-360	Initial OCI	227.65 ± 26.58	190-270
One-Hour OCI	235.29 ± 54.67	130-330	One-Hour OCI	240.00 ± 29.37	170-290
One-Day OCI	227.65 ± 62.40	150-330	One-Day OCI	221.76 ± 31.67	170-290

Examiner 1

ICC values were 0.84 and 0.79 for RCPM, 0.84 and 0.81 for RCPMJ, and 0.86 and 0.77 for OCI at the one-hour and one-day repetitions, respectively. SEM values for Examiner 1 were 11.63 and 13.92 mm² for RCPM, 10.30 and 7.91 mm² for RCPMJ, and 22.84 and 34.61 mm² for OCI at the one-hour and one-day repetitions, respectively (Table 3).

**Table 3:** ICC, SEM, and MDC values for Examiner 1 at one-hour and one-day repetitions RCPM: rectus capitis posterior minor; RCPMJ: rectus capitis posterior major; OCI: obliquus capitis inferior; ICC: intra-class correlation coefficient; SEM: standard error of measurement; MDC: minimal detectable changes

Muscle	Cronbach's Alpha	Single Measures ICC	Average Measures ICC	SEM (mm²)	MDC (mm²)	95% CI (Lower Bound)	95% CI (Upper Bound)
RCPM initial*1H	0.847	0.734	0.847	11.63	32.26	0.405	0.895
RCPMJ Initial*1h	0.842	0.728	0.842	10.30	28.55	0.394	0.892
OCI Initial*1h	0.698	0.536	0.698	35.61	98.71	0.09	0.803
RCPM initial*1D	0.797	0.663	0.797	13.92	38.60	0.282	0.863
RCPMJ Initial*1D	0.818	0.692	0.818	7.91	21.93	0.33	0.876
OCI Initial*1D	0.894	0.809	0.894	22.84	63.33	0.549	0.926

The overall average of three measurements was used to calculate the ICC for each muscle measured by Examiner 1 (Table 4). The ICC values were 0.85 for both RCPM and OCI, and 0.88 for RCPMJ, respectively. The corresponding SEM values were 14.62 mm² for RCPM, 10.54 mm² for RCPMJ, and 30.30 mm² for OCI.

**Table 4:** Overall ICC, SEM, and MDC values for Examiner 1 The table displays the intra-class correlation coefficient (ICC), the standard error of measurement (SEM), and minimal detectable changes (MDC) values, which are calculated based on the overall average of three measurements for Examiner 1. RCPM: rectus capitis posterior minor; RCPMJ: rectus capitis posterior major; OCI: obliquus capitis inferior

Muscle	Cronbach's Alpha	Single Measures ICC	Average Measures ICC	SEM (mm²)	MDC (mm²)	95% CI (Lower Bound)	95% CI (Upper Bound)
Overall RCPM	0.852	0.658	0.852	14.62	40.52	0.401	0.844
Overall RCPMJ	0.883	0.715	0.883	10.54	29.22	0.483	0.874
Overall OCI	0.855	0.664	0.855	30.30	84.00	0.409	0.847

Examiner 2

ICC values for Examiner 2 (Table 5) were 0.86 and 0.77 for RCPM values, 0.77 and 0.86 for RCPMJ values, and 0.61 and 0.64 for OCI values at the one-hour and one-day repetitions, respectively. SEM values for Examiner 2 were 11.82 and 13.40 mm² for RCPM, 11.91 and 7.04 mm² for RCPMJ, and 39.02 and 37.73 mm² for OCI.

**Table 5:** ICC, SEM, and MDC values for Examiner 2 at one-hour and one-day repetitions RCPM: rectus capitis posterior minor; RCPMJ: rectus capitis posterior major; OCI: obliquus capitis inferior; ICC: intra-class correlation coefficient; SEM: standard error of measurement; MDC: minimal detectable changes

Muscle	Cronbach's Alpha	Single Measures ICC	Average Measures ICC	SEM (mm²)	MDC (mm²)	95% CI (Lower Bound)	95% CI (Upper Bound)
RCPM initial*1H	0.862	0.757	0.862	11.82	32.78	0.448	0.905
RCPMJ Initial*1h	0.778	0.636	0.778	11.91	33.03	0.24	0.851
OCI Initial*1h	0.614	0.443	0.614	39.02	108.16	-0.031	0.755
RCPM initial*1D	0.778	0.636	0.778	13.40	37.14	0.239	0.851
RCPMJ Initial*1D	0.861	0.756	0.861	7.04	19.52	0.445	0.904
OCI Initial*1D	0.648	0.479	0.648	37.73	104.60	0.014	0.774

The overall average of three measurements was used to calculate the ICC for each muscle measured by Examiner 2. For Examiner 2, ICC values were 0.87 for RCPM, 0.81 for RCPMJ, and 0.70 for OCI. SEM values were 12.18 mm², 12.66 mm², and 38.91 mm² for RCPM, RCPMJ, and OCI, respectively. These data are available in Table 6.

**Table 6:** Overall ICC, SEM, and MDC values for Examiner 2 This table displays the intra-class correlation coefficient (ICC), the standard error of measurement (SEM), and minimal detectable changes (MDC) values calculated based on the overall average of three measurements for Examiner 2. RCPM: rectus capitis posterior minor; RCPMJ: rectus capitis posterior major; OCI: obliquus capitis inferior

Muscle	Cronbach's Alpha	Single Measures ICC	Average Measures ICC	SEM (mm²)	MDC (mm²)	95% CI (Lower Bound)	95% CI (Upper Bound)
Overall RCPM	0.875	0.699	0.875	12.18	33.77	0.459	0.866
Overall RCPMJ	0.811	0.589	0.811	12.66	35.09	0.311	0.807
Overall OCI	0.707	0.446	0.707	38.91	107.86	0.148	0.72

Inter-rater reliability

The inter-rater ICC values between Examiner 1 and Examiner 2 were 0.77 for RCPM, 0.82 for RCPMJ, and 0.68 for OCI. The SEM values were 13.74 mm² for RCPM, 10.67 mm² for RCPMJ, and 99.88 mm² for OCI. These data are available in Table 7.

**Table 7:** Inter-rater ICC, SEM, and MDC values RCPM: rectus capitis posterior minor; RCPMJ: rectus capitis posterior major; OCI: obliquus capitis inferior; ICC: intra-class correlation coefficient; SEM: standard error of measurement; MDC: minimal detectable changes

Muscle	Cronbach's Alpha	Single Measures ICC	Average Measures ICC	SEM (mm²)	MDC (mm²)	95% CI (Lower Bound)	95% CI (Upper Bound)
Overall RCPM	0.772	0.629	0.772	13.74	38.09	0.227	0.847
Overall RCPMJ	0.829	0.708	0.829	10.67	29.58	0.358	0.883
Overall OCI	0.688	0.525	0.688	36.03	99.88	0.075	0.797

**Figure 2:**
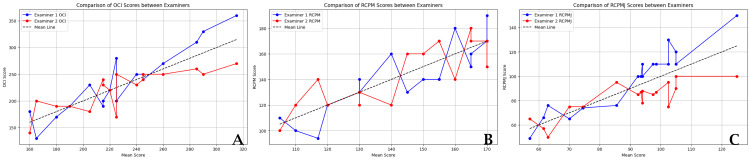
Comparison of Examiner’s CSA measurement values This chart displays the individual scores of each examiner for cross-sectional area (CSA) measurements of (A) OCI, (B) RCPM, and (C) RCPMJ. The x-axis represents the mean score, calculated as the average of scores provided by both examiners for each measurement. The y-axis represents the scores given by each examiner, illustrating their assessment relative to the overall average. RCPM: rectus capitis posterior minor; RCPMJ: rectus capitis posterior major; OCI: obliquus capitis inferior

**Figure 3:**
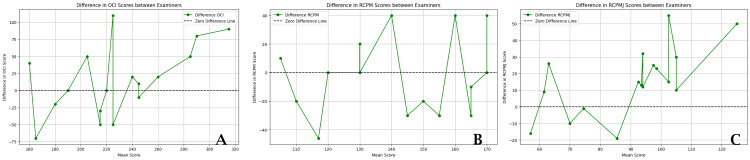
Comparison of Examiner’s CSA measurement values This chart depicts the differences between the examiners' scores for cross-sectional area (CSA) measurements of (A) OCI, (B) RCPM, and (C) RCPMJ. The x-axis represents the mean score, calculated as the average of scores provided by both examiners for each measurement. The y-axis represents the disparity between the scores of the two examiners, highlighting the consistency or variability in the scoring between examiners. RCPM: rectus capitis posterior minor; RCPMJ: rectus capitis posterior major; OCI: obliquus capitis inferior

Below is a comparative chart of examiner measurements, based on the average values from three measurements. The charts showcase examiner scores, with the x-axis representing the mean score averaged from the scores provided by both examiners for each measurement. The y-axis displays either the individual scores of each examiner (Figure 2) or the difference between their scores (Figure 3). This mean score on the x-axis serves as a baseline, facilitating the visualization of how each examiner's scores compare with each other and the overall average. This arrangement is designed to highlight the consistency or variability in scoring across different examiners.

ICC values less than 0.5 indicate poor reliability, values between 0.5 and 0.75 suggest moderate reliability, values between 0.75 and 0.9 indicate good reliability, and values greater than 0.9 reflect excellent reliability [21].

## Discussion

The Cronbach's alpha values indicate generally good reliability for the measurements of RCPM and RCPMJ across both examiners, with values consistently above 0.79, suggesting reliable internal consistency.

Examiner 1's measurements were reliable for RCPM and RCPMJ, with ICC values showing strong consistency over time. This suggests that Examiner 1's methods are dependable and could set the standard for best practices when measuring these muscles with sonography. Examiner 2 also demonstrated good reliability, although their results varied slightly more when measuring OCI. This might stem from differences in experience. It could also reflect how hard it is to measure OCI, given its location, or how the subject's muscles can change during the day due to emotional and physical modifications. Regarding SEM and MDC results, the SEM figures corresponded with the ICC patterns, providing a precision measure that complements the reliability indicators. The lower SEM values for RCPMJ and RCPM with both examiners indicate that these muscle groups are easier to measure consistently. The higher MDC values for OCI observed with Examiner 2 emphasize the difficulty in detecting small but clinically significant differences. This is crucial in medical settings, where small changes in muscle size or composition can influence treatment decisions or aid in diagnosis.

The inter-rater ICC values show good consistency between Examiner 1 and Examiner 2 for RCPM and RCPMJ, with ICC values of 0.772 and 0.822, respectively. This suggests that both examiners are relatively aligned in their measurement techniques for these two muscles, demonstrating reliable assessments across different evaluators.

In contrast, the ICC value for OCI is 0.688, indicating moderate consistency. This lower value indicates that there are more significant discrepancies between the two examiners when measuring the OCI muscle. The variability could stem from the muscle's complex anatomy, differences in examiner techniques, or the challenges of sonographic visualization of this specific muscle group. It suggests a need for further standardization in measurement protocols or additional training specifically focused on the OCI measurements to enhance consistency between different raters.

A comparative analysis of the CSA of the OCI with Cho's (2010) study, which was conducted similarly to ours, reveals consistency between Examiner 2's ICC and the ICC reported by Cho et al. (0.70 vs. 0.73). In contrast, Examiner 1 demonstrated a higher ICC (0.85 vs. 0.73). The mean CSA reported by the authors was lower compared to our examiners' measurements (mean ±SD for Examiner 1: 232.33±59.27 mm²; mean ±SD for Examiner 2: 229.8±29.20 mm² vs. mean ±SD from the compared study: 186±51 mm²), which may be attributed to differences in age ranges between the studies [10].

Comparative analysis of ICC, SEM, and MDC measurements for the RCPM muscle, referencing Ahmadipoor's (2021) study, which utilized the echo intensity tool of a sonographic device, reveals significantly lower values compared to our study. Ahmadipour reported ICC: 0.48, SEM: 4.98 mm², and MDC: 9.13 mm². In contrast, our measurements for Examiner 1 and Examiner 2 were ICC: 0.85 and 0.87, SEM: 14.62 mm² and 12.18 mm², and MDC: 40.52 mm² and 33.77 mm², respectively. These findings suggest that using echo intensity may be a less reliable but more precise method for measuring RCPM muscle properties compared to CSA sonogram measurement [22].

Comparing ICC results with Øverås' (2017) study, which measured the thickness of the RCPM muscle, reveals consistency between studies. Øverås reported an ICC (95% CI) of 0.86 (0.75-0.93), which is consistent with the ICCs of 0.87 and 0.85 for Examiner 1 and Examiner 2, respectively, in our study [23]. Overall, there appears to be a consistent agreement between previous studies and our reliability study regarding the suboccipital muscles.

Limitations

The study's limitations include its potential susceptibility to day-to-day variations in muscle physiology, which might affect measurements. Additionally, the psychological state of subjects, such as stress or fatigue, could influence muscle conditions, thereby impacting sonographic assessments.

Future research should focus on larger sample sizes and include a diversity of examiners to validate and possibly enhance the generalizability of these findings. Further research should also explore the impact of different sonographic equipment or settings to better understand the variability in measurements, particularly for OCI.

Future clinical implications

Standardization of Measurement Protocols

The findings suggest that rehabilitative sonography can reliably measure the CSA of RCPM and RCPMJ. However, the moderate consistency in measurements of the OCI highlights the demand for clear, standardized guidelines for probe positioning, patient posture, and scanning techniques to help reduce inter-examiner variability and enhance clinical utility.

Training and Skill Development

The differences in reliability between Examiners 1 and 2, particularly when measuring OCI, indicate that experience plays a significant role in obtaining accurate measurements. incorporated targeted training programs focusing on challenging anatomical regions like OCI can improve sonographer consistency.

Utility in Clinical Diagnosis and Treatment Planning

The strong intra-rater and inter-rater reliability for RCPM and RCPMJ suggest that rehabilitative sonography could be confidently used in clinical settings to assess and monitor the health and function of deep suboccipital muscles. Clinicians could use this tool to track muscle changes over time in patients with conditions such as neck pain, cervicogenic headaches, or postural imbalances, aiding in diagnostic precision and individualized treatment plans.

## Conclusions

This study underscores the reliability of sonographic measurements of the suboccipital muscles, with generally high reliability noted for RCPM and RCPMJ. However, the variability observed in OCI measurements, despite having moderate consistency, highlights the need for enhanced techniques and standardized protocols to ensure consistent evaluations across different examiners. These findings pave the way for improving diagnostic accuracy and treatment efficacy in clinical settings.
